# Probable clozapine-induced parenchymal lung disease and perimyocarditis: a case report

**DOI:** 10.1186/s12888-016-1158-1

**Published:** 2016-12-08

**Authors:** Erlend Bugge, Trygve Nissen, Rolf Wynn

**Affiliations:** 1 Division of Mental Health and Addictions, University Hospital of North Norway, Tromsø, Norway; 2 Department of Clinical Medicine, Faculty of Health Sciences, University of Tromsø, Tromsø, Norway

**Keywords:** Clozapine, Case report, Side effects, Pericarditis, Perimyocarditis, Diffuse parenchymal lung disease, Schizophrenia

## Abstract

**Background:**

Clozapine is the archetypical atypical antipsychotic, its primary indication being treatment resistant schizophrenia. Severe side effects caused by clozapine, including leukopenia, agranulocytosis, and myocarditis, are well known. A rarely described side effect is concurrent perimyocarditis and parenchymal lung disease.

**Case presentation:**

A previously physically healthy 23-year-old male Caucasian that suffered from schizophrenia presented with flu-like symptoms 1 week after starting clozapine treatment. Treatment with clozapine was discontinued. He developed respiratory distress. Investigations showed significant parenchymal infiltration in both of the lungs, pericardial fluid, and heart failure. He initially received treatment for suspected malignant neuroleptic syndrome and later for suspected infection, but these tentative diagnoses were not confirmed. The patient’s condition gradually improved. In retrospect, clozapine-induced parenchymal lung disease and perimyocarditis were deemed the most probable causes.

**Conclusions:**

Concurrent perimyocarditis and parenchymal lung disease are rare side effects of clozapine. Clozapine-induced disease in general is considered an exclusion diagnosis. Lacking a verifiable diagnosis when suspecting a side effect of clozapine, clinicians might treat the most likely and serious condition presenting and consider discontinuing clozapine until the diagnostic uncertainty is reasonably resolved.

## Background

Clozapine is the archetypical atypical antipsychotic, its primary indication being treatment resistant schizophrenia. The risk of leukopenia and agranulocytosis in clozapine treatment is well established, affecting approximately 3.0 and 0.5% of patients, respectively [1, 2]. Accordingly, all patients should routinely undertake blood samples. Clozapine induced myocarditis is a recognized, though perhaps overlooked, adverse effect of clozapine. Twenty years ago the prevalence was estimated at less than 0.1%, but increased awareness and research has revealed that the actual prevalence may be as high as 3.0% [3, 4]. The frequency of pericarditis caused by clozapine is yet to be established and we are aware of less than 10 cases that have been reported [5–7]. However, comorbid pericarditis and myocarditis precipitated by the same aetiological factors (drugs, virus, ischemia, etc.) and with common pathogeneses is not unusual. Given the prevalence of clozapine-induced myocarditis, the prevalence of pericarditis as an adverse effect of clozapine might be higher than the number of published case reports indicates. Clozapine-induced lung disease seems to be exceptional, given the fact that only a handful of cases have been published [8–10]. On the other hand, there might be a lesson to learn from the steady increase in reports of drug-induced lung disease in general. In 1972, only 19 drugs were known to cause lung disease [11]. Five years ago, the number had risen to approximately 350, whereas today more than 600 drugs are recognized as potentially pneumotoxic [12]. In the early 1960s, the first reports of pulmonary damage caused by tricyclic antidepressants were published, whereas today most psychotropic drugs are considered potentially pneumotoxic [13]. Case reports serve important functions, including reporting new or rare findings that may be of importance to clinicians and others [14–16]. Treatment with clozapine can result in a myriad of side effects and symptoms, often making clinical assessment difficult. This is even more the case when several organ systems are involved and the suspected conditions are considered highly infrequent. This case report demonstrates the challenges facing clinicians in such a case.

## Case presentation

**Fig. 1:**
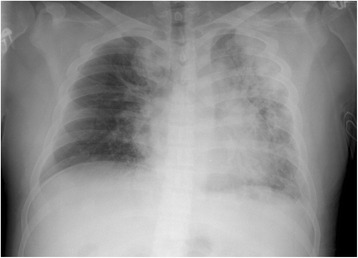
Chest x-ray. Chest x-ray showing massive diffuse parenchymal infiltration, predominantly of the left lung

A 23-year-old Caucasian man, diagnosed with schizophrenia and without any prior history of cardiac or pulmonary disease, was transferred from a psychiatric ward to an intensive care unit with flu-like symptoms, i.e., fever (39.7–40.2 °C), nausea, non-productive cough, headache, and muscle pain. The symptoms had developed approximately 1 week after monotherapeutic clozapine-treatment was initiated, and at the time of admittance the daily dose was 300 mg. Initial blood sampling demonstrated elevated levels of creatine kinase (2948 IU/L, range 50–400), C-reactive protein (154 mg/L, normal value <5), lactate dehydrogenase (359 IU/L, range 105–205), aspartate amino transferase (76 IU/L, range 15–45), erythrocyte sedimentation rate (24 mm/h, normal value <13), and myoglobin (248 μg/L, range <100), whereas white blood cells were in the normal range (9.9 × 10^9^/L, range 4–11). Blood and urine cultures were negative. Saturation of peripheral oxygen (SpO_2_) was 97%. Clinical examination revealed an elevated systolic blood pressure (178/75 mmHg) and tachycardia (120–130/min), but was otherwise normal, as were chest x-ray and electrocardiography (ECG). The patient had no obvious mental symptoms or autonomic instability. Despite the clinical picture being non-characteristic, malignant neuroleptic syndrome was suspected. Hence, clozapine was discontinued and treatment with oral bromocriptine (a dopamine agonist) was commenced with a dose of 2.5 mg three times a day. Clinically, the patient’s overall condition worsened in the next 24 h, and he reported dyspnoea and respiratory-dependent chest discomfort. Saturation of peripheral oxygen dropped to 88%. Troponin T was now elevated (0.41 μg/L, normal value < 0.03), as were fibrinogen (5.8 g/L, range 2–4), D-dimer (3.7 FEUμg/mL, normal value <0.5), and white blood cells (11.1 × 10^9^/L), with a mild eosinophilia (0.46 × 10^9^/L, range 0.04–0.4). On the other hand, creatine kinase had fallen (997 IU/L) and myoglobin had normalised (46 μg/L). Extensive testing for various infectious agents, such as streptococcus, coxsakcie A and B virus, Epstein-Barr virus, adenovirus, influenza A and B virus, parainfluenza 1 and 3 virus, parvo B19 virus, mycoplasma pneumoniae, chlamydia pneumoniae and cytomegalovirus, were undertaken without any pathogen identified. Likewise, repeated blood and urines cultures were negative. Given the fact that the patient had been in intramural psychiatric care for 2 months prior to the debut of his somatic symptoms, causes like exposure to toxins, substance abuse and other (prescribed or non-prescribed) drugs were believed to be unlikely. At examination, the patient had tachycardia (129–140/min), fever (39.7 °C) and prolonged exhalation with crepitation and diminished respiratory sounds over the lungs. The chest x-ray revealed significant parenchymal infiltration in both lungs (Fig. 1).

Echocardiography depicted a left ventricular ejection fraction of 54% (a.m. Simpson) and pericardial fluid. Notwithstanding the lack of a demonstrable infective agent and inconclusive blood samples, an infection was presumed. Accordingly, malignant neuroleptic syndrome was considered less likely. Thus, bromocriptine was suspended and intravenous antibiotic treatment with a cephalosporin, cefotaxime, started, with a daily dose of 6000 mg. The next couple of days there was little clinical change, but troponin T (0.20 μg/L) and white blood cells returned to normal, though still with a relative increase in eosinophils: 3.02 and 3.54% at day 3 and 4, respectively, compared to 0.50% the day before admittance and 0.56% at the day of admittance. C-reactive protein remained high (198–201 mg/mL). Echocardiography now exhibited biventricular hypokinesia and dilatation with mitral and tricuspid valve insufficiency, a left ventricular ejection fraction of 34% (a.m. Simpson), and increased pericardial fluid. Chest x-ray displayed regression of the parenchymal infiltration in both lungs. In the following week, there was a gradual improvement in the condition of the patient. The fever dissipated, his breathing became unrestricted and subjectively the patient felt much better. Correspondingly, the blood samples and chest x-rays normalized. Hence, after 8 days of treatment, the intravenous cephalosporin was terminated and the patient transferred back to the psychiatric ward. Repeated follow-ups during the next 4 years demonstrated blood samples within normal range and chest x-rays without identifiable pathology. Echocardiographies, however, showed a persistent cardiomyopathy with left ventricular hypokinesia and a left ventricular ejection fraction (a.m. Simpson) of 42–45%.

## Discussion

Adverse effects of clozapine can present with a multitude of symptoms, thus mimicking a wide array of conditions, as partially demonstrated by our case report. Even serious side effects may have an insidious onset, making early detection challenging. This is complicated by the fact that the physical health of patients with severe mental disorders often is poor, and comorbid somatic conditions such as obstructive lung disease and cardiovascular disease are common [17, 18]. At that, these patients are less likely to report somatic symptoms [19]. As a general rule, clozapine should always be suspected as a potential culprit when a patient develops new or worsened physical symptoms, in particular when the symptoms point to a systemic process and appear in the early stages of treatment. Most cases of myocarditis, pericarditis and lung disease seem to develop within 2 to 3 weeks of commencing clozapine [20]. It should also be taken into account that the concomitant use of psychotropic drugs adds to the risk of developing serious adverse effects, as does comorbid metabolic syndrome, heart and lung disease [21–25].

In this case, the diagnostic assessment was challenging. Throughout the process, there was a certain level of diagnostic doubt. Several diagnoses were deliberated, clozapine-induced disease included. The latter, however, has no pathognomonic clinical, biochemical, physical, radiographic or histologic markers. In our case, the clinicians could not rule out an infection and consequently chose the safer option: antibiotic treatment. Apparently, the patient seemed to respond to this treatment, but testing for an infection did not identify an infective agent. In retrospect, we did a thorough clinical review of the case in collaboration with cardiologists and pulmonologists. As a result, parenchymal lung disease and perimyocarditis were deemed the most likely diagnoses. Specifically, the temporality of the drug treatment and the clinical development, combined with the biochemical, radiological, and echocardiographic findings strongly indicated adverse effects of clozapine as causative. This hypothesis was strengthened when the Naranjo Adverse Drug Reaction Probability Scale estimated the causation as probable [26]. One lesson to take home from this case report might be that clozapine-induced disease is an exclusion diagnosis. Accordingly, lacking a verifiable diagnosis, the clinician should treat the most likely and potentially dangerous condition at any given moment, at the same time discontinue clozapine until diagnostic uncertainty is reasonably resolved.

Several investigations are relevant when suspecting a clozapine-related lung and/or heart condition. Introductorily, it should be noted that reports indicate that inflammation may precipitate clozapine toxicity, possibly due to a cytokine-mediated inhibition of cytochrome P450 1A2 [27]. Consequently, even moderate doses of clozapine could produce significant increase in serum levels in the presence of inflammation.

Elevated levels of C-reactive protein (incl. high-sensitivity C-reactive protein), erythrocyte sedimentation rate and plasma viscosity, are non-specific markers of inflammation. So are white blood cells, but despite the dominant hypothesis of a type I Ig E-mediated acute hypersensitivity reaction, there is no specific level or pattern of white blood cells signalling an adverse effect of clozapine. In other words, eosinophilia, or any other white blood cell pattern, may or may not be present [28]. On the other hand, the presence of eosinophilia in peripheral blood does make the diagnosis of a clozapine-related heart condition more likely, as demonstrated in the majority of documented cases [29]. Markers of myocardial damage such as myocardial muscle creatine kinase, myoglobin and troponins may be useful, but do not represent an unequivocal identification of myocarditis, nor pericarditis. Troponins, for instance, have a high specificity for myocardial damage, but the sensitivity for acute myocarditis and pericarditis is approximately 50 and 30%, respectively [30, 31].

Biopsy of heart and lung tissue may identify ongoing inflammation, but sampling error and interobserver variability in sample interpretation remain significant limitations to its diagnostic value. Furthermore, there is a certain risk of complications [32–35]. Hence, biopsying is not routinely undertaken when clozapine-triggered disease is suspected, but may be necessary in order to home in on the diagnosis. For instance, transbronchial biopsy may aid in the diagnosis of other pulmonary diseases such as pneumonia, sarcoidosis, etc. The same goes for bronchoalveolar lavage. It is especially helpful in differential diagnostics, primarily by excluding infective aetiology to pulmonary infiltrates, secondarily to demonstrate drug-induced eosinophilic pneumonia.

The ECG is widely used as a screening tool for cardiac pathology, despite its low sensitivity.

There are no characteristic ECG patterns in myocarditis and findings range from non-specific T-wave and ST-segment changes, to ST-segment elevation mimicking myocardial infarction, as well as supraventricular and ventricular arrhythmias, QTc prolongation, abnormal QRS configuration, etc. [36]. On the other hand, ECG, as well as echocardiography, could be inconspicuous in the presence of low-grade inflammation of the heart. Nevertheless, they are simple, low-risk procedures and ECG is generally recommended as a standard procedure before and during clozapine-treatment.

Cardiovascular magnetic resonance (CMR) enables detection of various non-specific features of myocarditis and pericarditis, though the sensitivity is variable, depending on the level of damage [37]. Nevertheless, CMR is preferable to chest x-ray and is the investigation of choice when myocarditis is presumed. Chest x-ray is typically obtained when drug-mediated pulmonary toxicity is suspected, but high-resolution computed tomography (HR-CT) is more sensitive than chest radiography for defining abnormalities [38, 39]. However, the sensitivity is time-dependent and repeated HR-CTs may therefore be necessary. For unknown reasons, HR-CT was not performed in this particular case, which in hindsight should have been undertaken in order to clarify the diagnosis. It is worth remembering that diffuse parenchymal lung changes is just one of many patterns that drug-induced lung reactions may assume [40]. Hence, radiological changes must be considered in conjunction with anamnestic, clinical and investigative information to determine the nature of pulmonary complications.

On pulmonary function tests, toxicity from most drugs result in a restrictive lung disease pattern, with a decrease in total lung capacity. Despite being sensitive to early changes in drug-induced lung function, these tests are unspecific and provide limited diagnostic information. Nonetheless, a pulmonary test could be useful in monitoring treatment.

In summary, when faced with the possibility of clozapine induced heart and/or lung disease, various and repeated investigative procedures are needed to make a qualified clinical assessment.

## Conclusions

In treatment-resistant schizophrenia, clozapine is often the drug of choice. However, the clinician should keep in mind the drug’s potentially deleterious side effects. Clozapine-induced diseases have no pathognomonic features, initially often masquerading as innocent infections such as the common cold or the flu. Even severe side effects might be difficult to pinpoint as clozapine related. In our case, the evidence of clozapine-induced lung disease does not seem as strong as the evidence of perimyocarditis. Besides discontinuing clozapine, no specific treatment has proven effective against adverse effects of clozapine. Thus, treatment is supportive and symptom-oriented. Myocarditis is an important risk factor for cardiomyopathy. Hence, follow-up is mandatory for all patients who have undergone myocarditis [41].

## Abbreviations

CMR: Cardiovascular magnetic resonance
ECK: Electrocardiogram
HR-CT: High resolution computed tomography
